# Selective Activation of the Infraspinatus During External Rotation Exercises in Participants with Rounded Shoulder Posture: Comparison of Three Common Exercises and Muscle Architecture-Based Exercise

**DOI:** 10.3390/medicina61020203

**Published:** 2025-01-23

**Authors:** Caglar Soylu, Emre Serdar Atalay, Bunyamin Haksever, Pervin Demir, Sinan Seyhan, Türker Bıyıklı

**Affiliations:** 1 Gülhane Faculty of Physical Therapy and Rehabilitation Ankara, University of Health Sciences, 06010 Ankara, Turkey; emreserdar.atalay@sbu.edu.tr; 2 Fit Level Wellness Center, 06510 Ankara, Turkey; yasbun@hotmail.com; 3 Faculty of Medicine, Basic Medical Sciences, Biostatistics and Medical Informatics, Ankara Yildirim Beyazit University, 06145 Ankara, Turkey; pervin.demr@gmail.com; 4 Faculty of Sport Sciences, Department of Coaching Education, Manisa Celal Bayar University, 45140 Manisa, Turkey; sinan.seyhan@cbu.edu.tr; 5 Departmentof Coaching Education, Marmara University Faculty of Sport Sciences, 34815 Istanbul, Turkey; turker.biyikli@marmara.edu.tr

**Keywords:** infraspinatus, muscle architecture, rounded shoulder posture, surface electromyography

## Abstract

*Background and Objectives*: The infraspinatus muscle is critical for shoulder stability and external rotation, yet achieving selective activation during exercises remains challenging. This study explores the effectiveness of a novel muscle architecture-based position (MABER) compared to traditional exercises in individuals with rounded shoulder posture. *Materials and Methods:* This prospective, cross-sectional study evaluated the selective activation of the infraspinatus during external rotation exercises in participants with rounded shoulder posture. Thirty-two participants (17 males and 15 females) were recruited. Electromyographic (EMG) activity of the middle trapezius, posterior deltoid, and infraspinatus muscles in the dominant limb was recorded during four exercise positions: standing external rotation (SER), side-lying external rotation (SDER), scapular plane external rotation (SPER), and MABER. *Results:* MABER produced the highest infraspinatus activity (*p* < 0.001), while the lowest activity was observed during SPER (*p* < 0.001). The SER position generated higher posterior deltoid activity compared to other positions (*p* < 0.001). The SDER position demonstrated the highest infraspinatus/posterior deltoid activity ratio (*p* < 0.001). *Conclusions:* The MABER position can be used as an effective exercise to strengthen the infraspinatus muscle. This study contributes to the literature by comparing MABER with traditional exercises and highlights its potential benefits for individuals with rounded shoulder posture.

## 1. Introduction

Resisted shoulder external rotation (ER) exercises are widely recognized as an effective intervention to strengthen the infraspinatus, the primary external rotator of the shoulder [1]. The infraspinatus plays a crucial role in stabilizing the humeral head within the glenoid fossa during glenohumeral joint motion. This stabilization is achieved through the synergistic activation of all rotator cuff muscles, which collectively ensure optimal joint mechanics and prevent dysfunction [2,3]. Proper activation of the infraspinatus is essential to mitigate common issues such as shoulder pain, instability, and subacromial irritation, particularly in populations with altered shoulder biomechanics or postural deviations.

Various studies have investigated exercise modifications to enhance infraspinatus activation while reducing engagement of non-target muscles, such as the deltoid. Sakita et al. [4] examined the effect of shoulder abduction angle on muscle activation during ER exercises, comparing 30° abduction to 0° abduction. Their findings indicated no significant changes in the activation of the infraspinatus, deltoid, or pectoralis major muscles across standing and side-lying positions, although posterior deltoid activity was higher at 30° abduction in both positions. This highlights the importance of exercise positioning in influencing muscle recruitment patterns. Minimizing deltoid activation is particularly significant as it reduces the superior migration of the humeral head, thereby alleviating subacromial pressure and irritation [4,5].

Research has also demonstrated that side-lying ER exercises result in greater activation of the infraspinatus and teres minor compared to standing positions. However, side-lying exercises also produce higher middle deltoid activation, which may detract from the targeted muscle engagement [1,6]. Horizontal abduction and ER exercises performed in the prone position have been reported to yield the highest infraspinatus activation [7]. Furthermore, studies comparing lateral recumbency ER exercises to other positions have found that the side-lying position maximizes infraspinatus activity while minimizing mid-trapezius and posterior deltoid engagement [6].

Despite these findings, the muscle architecture-based ER (MABER) position—defined as 25° abduction and 20° ER—has not been adequately explored in terms of its electromyographic response. Ward et al. [8] reported that rotator cuff muscles collectively achieve their maximum force-generating potential in this position, as sarcomere lengths align within the optimal range of 2.6–2.8 µm. This suggests that the MABER position offers a biomechanically advantageous posture for optimizing muscle activation. However, the lack of empirical data on its effectiveness in clinical or rehabilitative contexts represents a significant gap in the literature. The focus on this position is essential, as it directly leverages muscle architecture to enhance the efficiency and specificity of infraspinatus activation during ER exercises.

Rounded shoulder posture (RSP) is a common postural deviation characterized by scapular protraction and internal rotation bias, which alters shoulder biomechanics and predisposes individuals to pain and dysfunction. Addressing this internal rotation bias by strengthening the infraspinatus is critical to restoring stability and reducing dysfunction. While studies have explored the biomechanics of ER exercises, few have examined their specific impact on individuals with RSP. This study seeks to fill this gap by evaluating the electromyographic response of the infraspinatus in the MABER position, comparing its effectiveness between individuals with RSP and those with normal posture.

This research emphasizes the importance of understanding the unique biomechanical advantages of the MABER position in clinical and rehabilitative settings. By demonstrating how this position aligns with optimal muscle architecture to enhance infraspinatus activation, this study aims to provide evidence for a novel and targeted approach to shoulder rehabilitation. Furthermore, by comparing the responses of individuals with RSP to those with normal posture, this study seeks to highlight the clinical significance of tailoring exercise interventions to specific postural deviations. These findings could inform the development of more effective, evidence-based protocols for addressing shoulder pain and dysfunction.

Based on these observations, the following hypotheses are proposed: (1) The infraspinatus muscle will demonstrate the highest activation during the muscle architecture-based external rotation exercise (MABER) compared to other commonly used external rotation positions. (2) Exercises performed in the MABER position will result in reduced activation of the middle trapezius and posterior deltoid muscles compared to other positions. (3) Individuals with RSP will exhibit distinct activation patterns in external rotation exercises compared to populations with normal posture, further emphasizing the importance of tailored exercise interventions.

## 2. Materials and Methods

### 2.1. Study Design and Participants

In this cross-sectional and quantitative study, 32 participants (17 males, 15 females) without shoulder pain or injury history were recruited, specifically targeting those with rounded shoulder posture (RSP). This study’s exclusion criteria included a history of shoulder injuries, fractures, luxations, cervical dysfunctions affecting upper limbs, and neurologic/musculoskeletal disorders impeding shoulder rotation. Sample size calculations were based on pilot data obtained from a group of 10 participants, using G*power software (version 3.1.9.7) aiming for significant statistical power and effect size. A priori calculation of the sample size was performed with a power of 0.80, an alpha level of 0.05, and an effect size of f = 0.40, classified as large (ηpartial^2^ = 0.924) based on the pilot data. The pilot study provided preliminary data on the variance and effect size observed in the dependent variables, justifying the parameters used in the calculation. This preliminary data demonstrated the feasibility of detecting very large effect sizes within the context of the current study design. However, the determination of a very large effect size (ηpartial^2^ = 0.924) necessitates clear documentation of the pilot study methodology and results to substantiate its representativeness and reliability for broader sample size estimation. Ethical compliance was ensured with informed consent from participants, approval from the University of Health Sciences Ethics Committee (2023/004), and registration in the ClinicalTrials.gov Protocol (NCT06070909), adhering to the Declaration of Helsinki.

### 2.2. Measuring Angle

**Figure 1:**
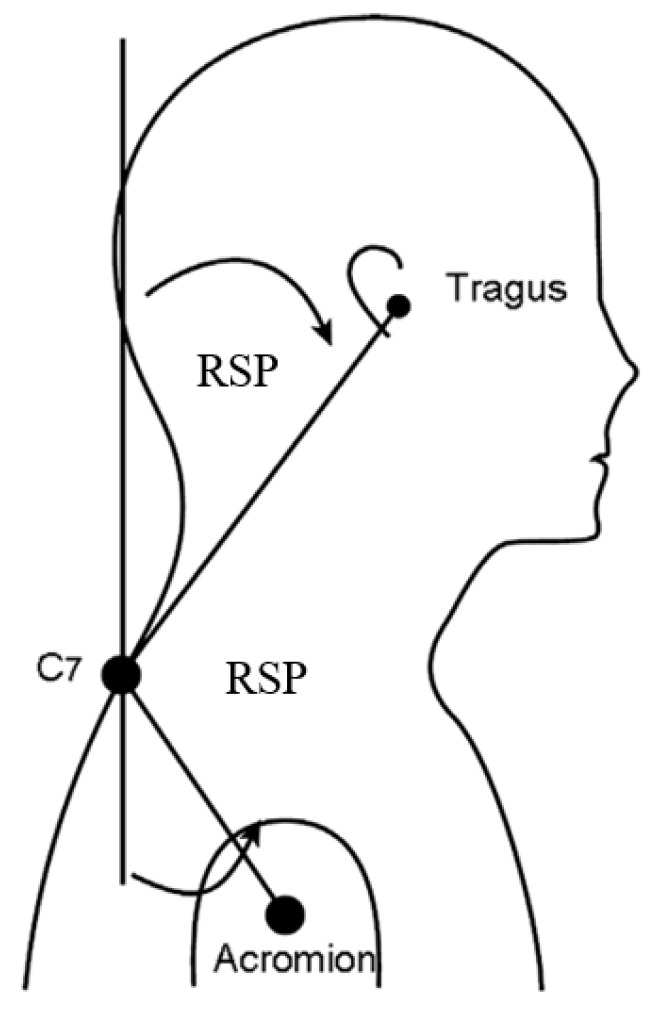
Rounded shoulder posture angle (RSP) measured from the vertical anteriorly to a line connecting the tragus and the C7 marker. Rounded shoulder posture angle (RSP) measured from the vertical posteriorly to a line connecting the C7 marker and the acromial marker [9].

The rounded shoulder posture (RSP) was measured using markers on the tragus, C7, and acromion, captured by two strategically placed cameras. Participants were photographed from side and rear views in a natural standing position, with instructions to maintain normal shoulder posture. Motion data were collected and analyzed using software to determine angles, specifically the rounded shoulder angle, which is the angle between the humerus and a line from its midpoint to the C7 spinous process. This study followed Thigpen et al. [9] in using 52° as the reference angle for RSP (Figure 1) [10,11].

### 2.3. EMG Measurements

Signal acquisition, processing, and analysis were performed using a Noraxon Ultium EMG sensor system (Noraxon USA, Inc., Scottsdale, AZ, USA). Signals were differentially amplified (CMRR > 100 dB; input impedance > 100 mΩ; gain 1000 dB, signal to noise ratio;1 μV RMS), and digitized at a sampling rate of 4000 Hz. Two filters were applied, including a band-pass filtered from 10 to 500 Hz using a first-order high-pass and fourth-order low-pass Butterworth filter to remove undesirable artefacts and a notch filter (60 Hz) to eliminate noise and the root mean square (RMS) was then calculated. A cancellation algorithm was applied to remove ECG signal contamination. The Noraxon MyoResearch XP program was used to process the data (version 3.16; Noraxon Inc., Scottsdale, AZ, USA). The data for each trial were expressed as a percentage of the calculated mean RMS of the maximal voluntary isometric contraction (MVIC) (%MVIC), and the mean %MVIC of 3 trials was used for analysis.

The electrode sites were cleansed with rubbing alcohol after shaving. After skin preparation, disposable Ag/AgCl bipolar surface electrodes with 2 cm inter-electrode distance (Noraxon Inc., Arizona, USA) were used to record the EMG from posterior deltoid (PD), middle trapezius (MT), and infraspinatus (IS) consistent with established guidelines (SENIAM and De Luca) [12]. For each subject, the dominant arm, which was defined as the upper extremity used to eat and write, was tested. Two surface electrode pairs were placed on the posterior deltoid (electrodes were located 2 cm below the lateral border of the spine of the scapula and angled obliquely to the arm), middle trapezius (midway on a horizontal line between the root of the spine of the scapula and the third thoracic spinous process), and the infraspinatus (electrodes were parallel to and approximately 4 cm below the spine of the scapula on the lateral aspect over the infrascapular fossa) [7].

For normalization, the RMS of a 5 s MVIC was measured three times for each muscle. The posterior deltoid was tested in a prone position with the arm abducted to 90° and in neutral rotation (palm down). Resistance was applied in an anterior direction proximal to the elbow. The infraspinatus was assessed while seated, with the elbow extended to 90° and the shoulder at 0° abduction and neutral rotation. To produce internal shoulder rotation, resistance was applied immediately above the wrist. To test the middle trapezius muscle, the shoulder was abducted and rotated externally with the elbow extended [7,13]. The maximum effort against manual resistance was used to hold each contraction for 5 s. The participants were given a two-minute break between each test to minimize muscle fatigue. For this study, participants underwent a supervised 1-Repetition Maximum (1RM) technique testing protocol, which included a standardized warm-up and progressive weight increments until the maximum achievable weight was identified within 3–5 attempts. This approach minimizes fatigue while ensuring precise measurements. The weight used for the exercises was then calculated as a percentage of the participant’s 1RM to ensure optimal resistance levels tailored to their strength capacity, enhancing both safety and the effectiveness of the intervention.

### 2.4. Procedures

**Figure 2:**
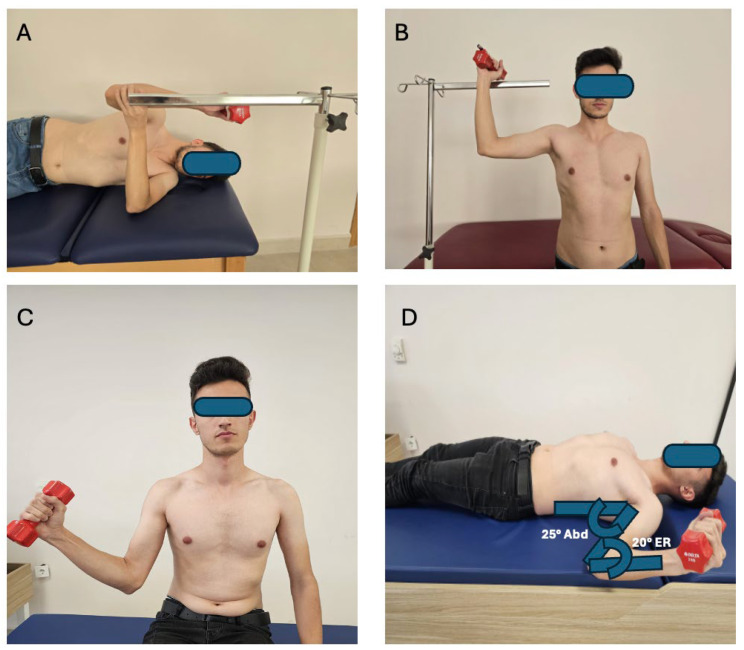
(**A**) Side-lying external rotation (SDER), (**B**) standing external rotation (SER), (**C**) standing ER in the scapular plane (SPER), (**D**) ER in muscle architecture-based position (MABER).

In this study, participants were first oriented about the experiment, followed by maximum voluntary isometric contraction (MVIC) assessments for specific muscles. Electromyography (EMG) activities were recorded during four exercises. Instructions and practice for each exercise were provided, with a target bar guiding the movement. Exercises included side-lying external rotation (SDER), standing external rotation (SER), standing ER in the scapular plane (SPER), and ER in muscle architecture-based position (MABER), each with distinct starting and ending positions as detailed by Ha et al. [6], Reinold et al. [1], and Ward et al. [8] (Figure 2). EMG data were collected over 10 s trials, regulated by a metronome. Exercise order was randomized with rest intervals, and dumbbell weights were determined for each participant. The infraspinatus/posterior deltoid muscle activity ratio was calculated for analysis.

### 2.5. Statistical Analyses

The methodology for statistical analysis in our study included summarizing variables as mean ± standard deviation and median with quartiles. Intra-rater reliability of MVIC percentages for each exercise was assessed using the Intraclass Correlation Coefficient (ICC), following the classification by Koo & Li. The ICC (ICC; two-way mixed effects, the mean of multiple measurements, absolute agreement) was calculated to evaluate the intra-rater reliability of the (%) MVIC measurements for each exercise. The following limits were used: <0.5: poor, 0.50–0.75: moderate, 0.76–0.90: good, and >0.90: excellent reliability [14]. Normal distribution and variance equality were checked using Shapiro–Wilk and Mauchly’s tests, respectively. Differences in exercises were analyzed using one-way ANOVA with repeated measures or Friedman’s ANOVA, depending on the assumption test results. Significant differences between exercises were further examined with Bonferroni-adjusted pairwise comparisons. Analyses were conducted in R Studio (2022.02.3) using specific packages [15], and statistical significance was set at *p* < 0.05.

## 3. Results

The mean age of the participants was 21.47 ± 0.92 years (median = 22.0; min = 20.0; max = 23.0), and the mean BMI was 21.58 ± 1.09 kg/m2 (median = 21.56; min = 20.10; max = 23.41). The 25 participants’ dominant side was right. The average RSP angle for the participants was 54.3 ± 1.8°. PD, IS, and MT muscle activity was measured during four different exercise positions. The intra-rater reliability values obtained in each position are summarized in Table 1.

**Figure 3:**
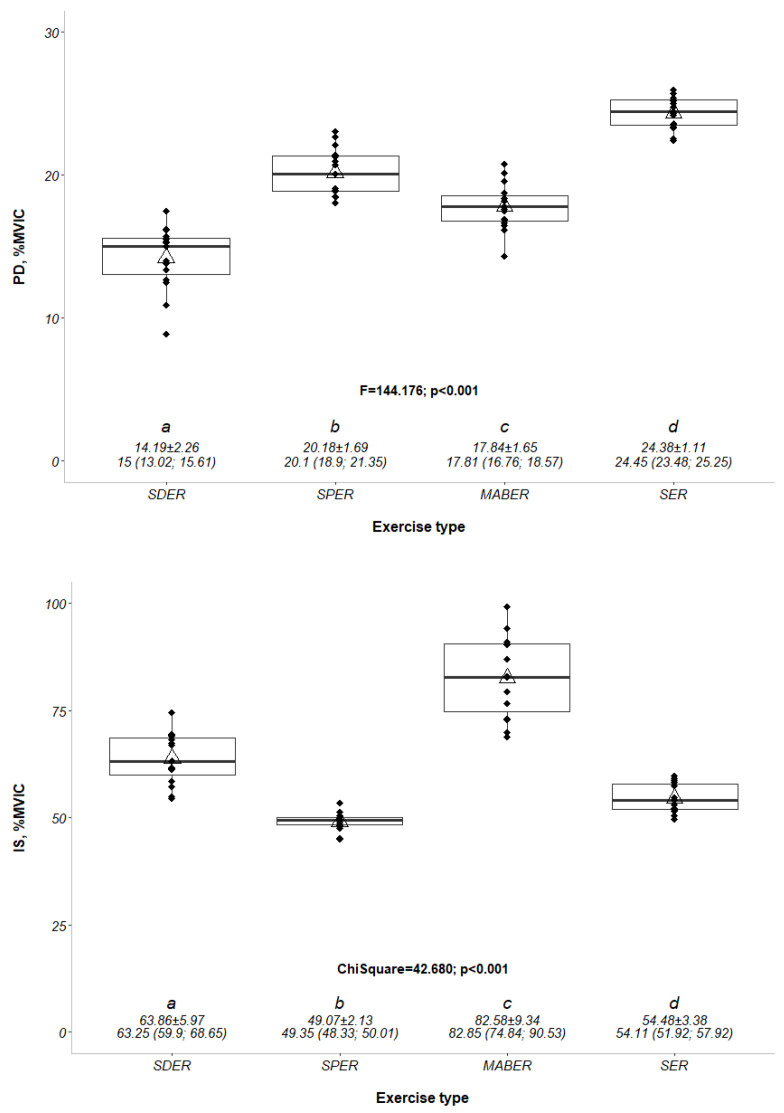
The percentage of maximum voluntary isometric contraction (%MVIC) values of the posterior deltoid (PD) and infraspinatus (IS) muscles across different external rotation exercise positions. PD: posterior deltoid, IS: infraspinatus, %MVIC: the percentage of maximal voluntary isometric contraction, SDER: side-lying external rotation, SPER: scapular-plane external rotation, MABER: muscle-architecture based external rotation, SER: standing external rotation. F-χ^2^; *p*: test statistic value and *p*-value of the repeated measures ANOVA within a factor-Friedman test.^a,b,c,d^: The differences are shown with lettering as a result of the pairwise comparisons made with the Bonferroni adjustment. The data were summarized as mean ± standard deviation and median (quartile 1; quartile 3). The y-axis of (1) was set to 0–30.

Means/ranks of muscle activities were significantly different in at least two exercise positions (*p* < 0.001). The normalized EMG activity of the infraspinatus and posterior deltoid muscle are shown in Figure 3 and Table 2. We observed the largest increase in muscle activity during exercise in the MABER (%82.58 ± 9.34) and the least activity in the SPER (%49.07 ± 2.13) (χ^2^ = 42.680; *p* < 0.001). The normalized EMG activity in the posterior deltoid was less in the SPER (%20.18 ± 1.69) than in the SER (%24.38 ± 1.11). Posterior deltoid EMG activity was less in the SDER (%14.19 ± 2.26) than the MABER (%17.84 ± 1.65) (F = 144.176; *p* < 0.001). The effect size (η^2^) calculations reveal substantial differences between exercise types for muscle activation. Specifically, the posterior deltoid (PD) showed a very large effect size (η^2^ = 0.92), indicating a significant impact of exercise type on its activation. Similarly, the infraspinatus (IS) demonstrated a large effect size (η^2^ = 0.78), highlighting considerable variability in muscle engagement across conditions. The middle trapezius (MT) exhibited the largest effect size (η^2^ = 0.95), further emphasizing the pronounced differences in activation. Lastly, IS_PD also showed a large effect size (η^2^ = 0.76), underscoring the notable influence of exercise type on muscle performance. These results confirm that exercise type has a significant and substantial effect on the activation of the studied muscles.

**Figure 4:**
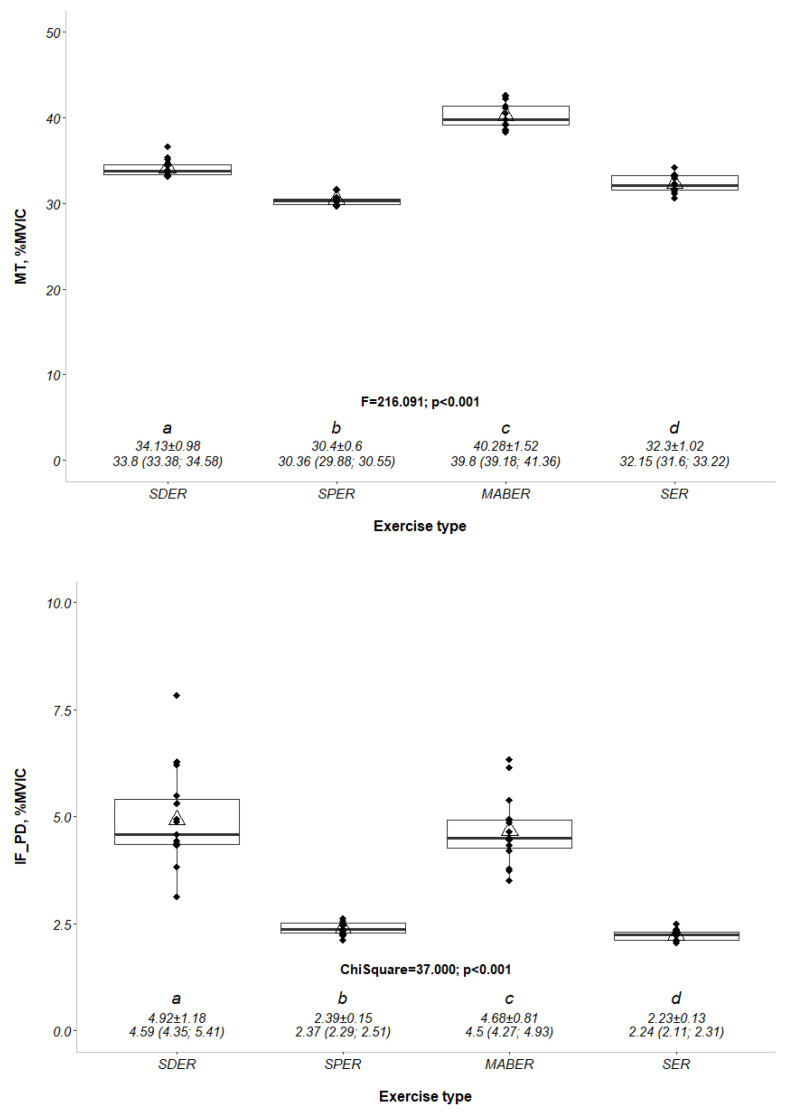
The percentage of maximum voluntary isometric contraction (%MVIC) values of the middle trapezius (MT) and infraspinatus-to-posterior deltoid ratio (IS_PD) muscles across different external rotation exercise types. MT: middle trapezius, IS_PD: infraspinatus/posterior deltoid, %MVIC: the percentage of maximal voluntary isometric contraction, SDER: side-lying external rotation, SPER: scapular-plane external rotation, MABER: muscle-architecture based external rotation, SER: standing external rotation. F-χ^2^; *p*: test statistic value and *p*-value of the repeated measures ANOVA within a factor-Friedman test.^a,b,c,d^: The differences are shown with lettering as a result of the pairwise comparisons made with the Bonferroni adjustment. The data were summarized as mean ± standard deviation and median (quartile 1; quartile 3). The y-axis of (1) and (2) was set to 0–50 and 0–10, respectively.

The normalized EMG activity in the middle trapezius was greater in the MABER than in the SDER, SER, and SPER (F = 216.091; *p* < 0.001) (Figure 4). The EMG ratio (infraspinatus/posterior deltoid) was significantly higher in SDER than in MABER, SPER, and SER (χ2 = 37.000; *p* < 0.001). The normalized EMG ratios of SDER, MABER, SPER, and SER were 4.92 ± 1.18, 4.68 ± 0.81, 2.39 ± 0.15, and 2.23 ± 0.13, respectively. The differences in muscle activity during each exercise position are shown in Figure 4 and Table 2.

## 4. Discussion

It is known that the infraspinatus muscle plays a crucial role in the nonoperative and postoperative management of rotator cuff tears, and numerous exercises have been developed to strengthen it based on EMG assessment of muscle activity [6]. The current trend in exercise programs is to emphasize functional restoration by strengthening specific target muscles with minimal involvement of nearby musculature [16]. The present study investigated the muscle activity of three muscles across four different exercise positions and evaluated the infraspinatus/posterior deltoid (IF/PD) ratio, which has significant clinical implications.

Researchers have long debated the most effective exercises for targeting the infraspinatus while minimizing activation of surrounding muscles. Previous studies have demonstrated high infraspinatus activity in prone and side-lying positions [6,16,17,18]. Similarly, Reinold et al. [1] emphasized the value of prone external rotation exercises, while Ha et al. [6] highlighted the side-lying position’s potential. Recent evidence suggests that combined external rotation and scapular retraction exercises improve scapular alignment and muscle activation in individuals with rounded shoulder posture (RSP), which is critical for targeted rehabilitation [19]. Additionally, exercises combining external shoulder joint rotation with contraction of the shoulder have been shown to significantly improve muscle activity, strength, and scapular alignment. A recent study by Moon et al. [20] demonstrated a significant increase in middle trapezius activity and external rotator strength, as well as reduced muscle tone and stiffness in individuals with RSP when external rotation exercises were combined with scapular contraction. These findings align with the present study’s results, emphasizing the importance of integrating external rotation into therapeutic interventions for individuals with RSP.

Our findings suggest that the muscle architecture-based external rotation exercise (MABER) is the most effective for infraspinatus strengthening, followed by the side-lying external rotation (SDER) and the standing external rotation (SER). The scapular plane external rotation (SPER) demonstrated the least effectiveness. These results suggest that MABER provides optimal activation of the infraspinatus muscle, supporting the hypothesis that it should be favored for targeted strengthening.

One of the limitations of this study is the lack of discussion regarding the role of gravity in the MABER position. It is important to clarify that gravity does not assist the movement in this position. Unlike positions where gravity might reduce the load (e.g., certain standing exercises), the MABER position leverages the biomechanical advantage provided by the muscle’s architecture, specifically its optimal length-tension relationship and lever arm mechanics. The increased muscle activity observed in the MABER position can be attributed to these factors rather than any reduction in resistance due to gravity. Ward et al. [8] emphasized that rotator cuff muscles achieve their maximum force-producing potential when the sarcomere length is in the optimal range, which coincides with the MABER position. Therefore, the heightened infraspinatus activation in this position is due to these biomechanical optimizations rather than an external reduction in load.

This study concludes that the MABER position is the most effective; however, it is also critical to explain the lower efficiency of other positions. The SPER, for example, demonstrated the least effectiveness due to its inherent biomechanical limitations, including suboptimal scapular positioning and reduced leverage for the infraspinatus. In contrast, the SER position produced the highest posterior deltoid activation (24% MVIC) at 90° abduction, aligning with previous studies by Reinold et al. [1] and Ha et al. [6]. This higher deltoid activation is likely due to the stabilization requirements of the upper extremity in this position. Although this can mimic functional shoulder movements in athletic activities, it also carries the risk of anterior humeral head translation and compensatory deltoid overactivation, as highlighted by Gasbarro et al. [21] and Gu et al. [22].

Interestingly, SDER showed the highest IS/PD ratio, with relatively lower posterior deltoid activation compared to other positions. This finding underscores its value as a starting point in rehabilitation protocols. The reduced posterior deltoid activity in SDER can be explained by the side-lying position’s reduction of gravitational influence, which minimizes compensatory activation. This makes SDER an ideal exercise for selectively targeting the infraspinatus while minimizing deltoid compensation, particularly in early rehabilitation stages.

MABER was also associated with increased middle trapezius (MT) activity, which supports scapular upward rotation. While this may enhance scapular stabilization, excessive MT activation could potentially reduce infraspinatus involvement by inducing scapular adduction. Despite this limitation, MABER remains a strong candidate for infraspinatus strengthening due to its ability to optimize muscle architecture and functional activation. Future studies should explore ways to balance MT activation while maintaining targeted infraspinatus strengthening.

This study had several limitations that must be addressed. Firstly, the results cannot be generalized to other populations, as the participants were limited to young individuals with RSP. Future research should include symptomatic and asymptomatic individuals across various age groups and pathologies, such as instability or subacromial impingement. Additionally, different types of resistance exercises (e.g., elastic bands) should be investigated. Secondly, this study did not stratify participants by the severity of RSP, which may influence muscle activation. Stratified analysis could provide deeper insights into the role of RSP severity in exercise outcomes. Thirdly, the use of surface EMG poses a risk of crosstalk from adjacent muscles. To minimize this limitation, future studies should include additional muscles, such as the serratus anterior and rhomboids, to better understand compensatory mechanisms. Finally, this study did not incorporate kinematic analysis to evaluate humeral and scapular movements. Future research using motion analysis systems could provide valuable insights into the dynamic interactions of the humerus, scapula, and shoulder musculature during external rotation exercises.

## 5. Conclusions

Our results suggest that the MABER is an effective exercise as it selectively activates the infraspinatus muscle while minimizing the activity of the posterior deltoid muscle in participants with rounded shoulder posture. These findings may help clinicians design effective exercise programs. Moreover, the findings present innovative approaches for the targeted activation of shoulder complex muscles during specific activities, which may be useful for use in training, injury prevention, and rehabilitation programs.

## Figures and Tables

**Table 1:** The intra-rater reliability coefficients (ICC) of measurements.

	Muscle		
	PD	IS	MT
MVIC	0.994	0.999	0.997
% MVIC during			
SDER	0.911	0.930	0.914
SPER	0.760	0.940	0.715
MABER	0.946	0.987	0.955
SER	0.872	0.990	0.819

PD: posterior deltoid, IS: infraspinatus, MT: middle trapezius, (%)MVIC: (the percentage of) maximal voluntary isometric contraction, SDER: side-lying external rotation, SPER: scapular-plane external rotation, MABER: muscle-architecture based external rotation, SER: standing external rotation.

**Table 2:** The comparison of muscle activity during the four different exercise types.

Exercise Type	%MVIC of Muscle			
	PD	IS	MT	IS_PD
SDER	14.19 ± 2.26 ^a^	63.86 ± 5.97 ^a^	34.13 ± 0.98 ^a^	4.92 ± 1.18 ^a^
SPER	20.18 ± 1.69 ^b^	49.07 ± 2.13 ^b^	30.40 ± 0.60 ^b^	2.39 ± 0.15 ^b^
MABER	17.84 ± 1.65 ^c^	82.58 ± 9.34 ^c^	40.28 ± 1.52 ^c^	4.68 ± 0.81 ^a^
SER	24.38 ± 1.11 ^d^	54.48 ± 3.38 ^b^	32.30 ± 1.02 ^d^	2.23 ± 0.13 ^b^
	F = 144.176; *p* < 0.001	χ^2^ = 42.680; *p* < 0.001	F = 216.091; *p* < 0.001	χ^2^ = 37.000; *p* < 0.001

PD: posterior deltoid, IS: infraspinatus, MT: middle trapezius, IS_PD: infraspinatus/posterior deltoid, (%)MVIC: (the percentage of) maximal voluntary isometric contraction, SDER: side-lying external rotation, SPER: scapular-plane external rotation, MABER: muscle-architecture based external rotation, SER: standing external rotation F; *p*: test statistic value and *p*-value of the repeated measures ANOVA within a factor ^a,b,c,d^: Pairwise comparisons using *t*-tests with pooled SD by Bonferroni adjustment method. ^a^: SDER (shoulder external rotation in side-lying position), ^b^: SPER (shoulder external rotation in standing position), ^c^: MABER (modified abduction and external rotation), ^d^: SER (shoulder external rotation in prone position).

## Data Availability

The data illustrated in the present study are available on reasonable request from the corresponding author.
